# Molecular Determinants and Specificity of mRNA with Alternatively-Spliced UPF1 Isoforms, Influenced by an Insertion in the ‘Regulatory Loop’

**DOI:** 10.3390/ijms222312744

**Published:** 2021-11-25

**Authors:** Monikaben Padariya, Robin Fahraeus, Ted Hupp, Umesh Kalathiya

**Affiliations:** 1 International Centre for Cancer Vaccine Science, University of Gdansk, ul. Kładki 24, 80-822 Gdansk, Poland; monikaben.padariya@ug.edu.pl (M.P.); robin.fahraeus@inserm.fr (R.F.); 2 Inserm UMRS1131, Institut de Génétique Moléculaire, Université Paris 7, Hôpital St. Louis, F-75010 Paris, France; 3 Department of Medical Biosciences, Umeå University, Building 6M, 90185 Umeå, Sweden; 4 Regional Centre for Applied Molecular Oncology (RECAMO), Masaryk Memorial Cancer Institute, Zlutykopec 7, 65653 Brno, Czech Republic; 5 Institute of Genetics and Cancer, University of Edinburgh, Edinburgh EH4 2XR, UK

**Keywords:** UPF1, GC-rich, AU-rich, isoform, regulatory loop, mRNA, PTC, molecular dynamics, degradation, stability, NMD, alternatively spliced, motifs

## Abstract

The nonsense-mediated mRNA decay (NMD) pathway rapidly detects and degrades mRNA containing premature termination codons (PTCs). UP-frameshift 1 (UPF1), the master regulator of the NMD process, has two alternatively-spliced isoforms; one carries 353-GNEDLVIIWLR-363 insertion in the ‘regulatory loop (involved in mRNA binding)’. Such insertion can induce catalytic and/or ATPase activity, as determined experimentally; however, the kinetics and molecular level information are not fully understood. Herein, applying all-atom molecular dynamics, we probe the binding specificity of UPF1 with different GC- and AU-rich mRNA motifs and the influence of insertion to the viable control over UPF1 catalytic activity. Our results indicate two distinct conformations between 1B and RecA2 domains of UPF1: ‘open (isoform_2; without insertion)’ and ‘closed (isoform_1; with insertion)’. These structural movements correspond to an important stacking pattern in mRNA motifs, i.e., absence of stack formation in mRNA, with UPF1 isoform_2 results in the ‘open conformation’. Particularly, for UPF1 isoform_1, the increased distance between 1B and RecA2 domains has resulted in reducing the mRNA–UPF1 interactions. Lower fluctuating GC-rich mRNA motifs have better binding with UPF1, compared with AU-rich sequences. Except CCUGGGG, all other GC-rich motifs formed a 4-stack pattern with UPF1. High occupancy R363, D364, T627, and G862 residues were common binding GC-rich motifs, as were R363, N535, and T627 for the AU-rich motifs. The GC-rich motifs behave distinctly when bound to either of the isoforms; lower stability was observed with UPF1 isoform_2. The cancer-associated UPF1 variants (P533L/T and A839T) resulted in decreased protein–mRNA binding efficiency. Lack of mRNA stacking poses in the UPF1_P533T_ system significantly decreased UPF1-mRNA binding efficiency and increased distance between 1B-RecA2. These novel findings can serve to further inform NMD-associated mechanistic and kinetic studies.

## 1. Introduction

Aberrant, misfolded, and mislocalized proteins are dangerous to cell viability due to their toxicity, which can be the cause of multiple human diseases such as Parkinson’s and Alzheimer’s diseases, frontotemporal dementia, cystic fibrosis, and several others [1,2,3,4,5,6,7]. Natural selection pressures have led to a cellular quality control pathway that prevents aberrant proteins at the ribosome or that senses the aberrations in the mRNA templates, resulting in their degradation. Many quality control or protective mechanisms are engaged cotranslationally, while proteins are being synthesized from mRNA [1,7,8]. Protein synthesis, along with the degradation of defective proteins, is an energetically demanding processes, and ribosome-associated quality control can hinder futile aberrant protein synthesis. The mRNA surveillance quality control pathways such as the nonsense-mediated mRNA decay (NMD) can detect as well as eliminate the defective mRNAs with premature termination codons (PTC), the no-go decay (NGD) pathway can trace truncated and stalled in translation mRNAs, and the non-stop decay (NSD) pathway can detect mRNAs without natural stop codons [7,9,10,11,12].

Particularly at the mRNA level, two essential features are observed: (i) either it contains the correct set of proteins bound to a particular mRNA, or (ii) the coding potential of the mRNA is intact. Here, the NMD pathway copes with the latter one; by degrading the PTC-containing mRNAs, it consequently reduces the accumulation of potentially toxic truncated proteins [10,13,14]. The NMD process is one of the best studied translation-dependent mRNA surveillance mechanisms, which typifies all eukaryotes examined to date and helps to regulate the quality of gene expression. Due to its capacity of detecting a PTC for accelerating the degradation of the aberrant mRNA, NMD is an important modulator of genetic disease phenotypes in humans [15,16]. In addition, one-third of inherited genetic diseases are caused by mRNAs harboring PTC as a result of nonsense mutations [15,16]. By degrading transcriptome, the NMD process can harbor distinct contexts at translation termination (e.g., uORF (upstream open reading frames)-containing mRNAs, long 3′UTRs), suggesting that it is a critical regulatory pathway [17,18,19,20,21,22]. In relation to this, the NMD pathway can exert a beneficial or a detrimental effect: the former if it prevents the synthesis of toxic truncated proteins and the latter if it prevents the production of proteins having some residual or partial functions [14,15,23,24,25].

**Figure 1:**
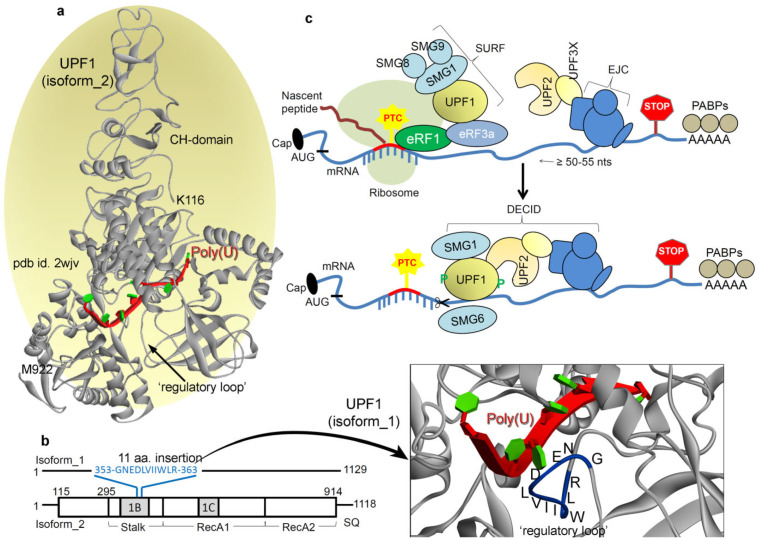
The master regulator of the NMD (nonsense-mediated mRNA decay) pathway: UPF1 (UP-frameshift 1). (**a**) Optimized UPF1 isoform_2 model structure [40] with the poly(U) mRNA motif, constructed based on the pdb (protein data bank) id: 2wjv [67] and 2xzo [37]. The crystal structure of UPF1 (pdb id: 2wjv [67]) is with the UPF2 protein, but since UPF1 adopts a different conformation when bound with UPF2, we removed it from the presentation. In addition, the poly(U) mRNA motif has been inserted into the structural representation (from pdb id: 2xzo [37]) as a reference since it is not present in the original crystal structure (pdb id: 2wjv [67]). (**b**) Isoform_1 structure of the UPF1 (build considering the pdb id: 2wjv [67]) protein, with the 11 aa (amino acids) insertion (353-GNEDLVIIWLR-363) in the ‘regulatory loop’. These 353-GNEDLVIIWLR-363 residues are highlighted over the protein structure in blue color. This UPF1 isoform_1 structure with inserting 11 aa was modeled considering the isoform_2 crystal structure as the template (pdb id: 2wjv) [67]. (**c**) In the NMD pathway, recognition of PTC-containing mRNA transcript requires UPF1, UPF2, and UPF3 proteins as the core machinery, along with various suppressors with morphogenetic effect on genitalia (SMG) proteins [7,61]. There is a general agreement that NMD substrate recognition relies upon differences in mRNA ribonucleoprotein (mRNP) composition between normal mRNAs and PTC-containing mRNA transcripts. In mammalian cells, an exon–exon junction complex (EJC) of proteins deposited at exon–exon boundaries during pre-mRNA splicing is considered to be a primary determinant of NMD [62]. Translation termination at a nonsense codon located ~50–55 nucleotides upstream of an exon–exon junction usually triggers NMD. It is suggested that translation termination events leading to NMD entail the SURF complex, consisting of SMG1, UPF1, eRF1 (eukaryotic translation termination factor 1), and eRF3 [41]. Once translation terminates, UPF1 then interacts with UPF2 that is bound to the EJC together with additional UPF3 proteins. These interactions result in the assembly of the surveillance complex, UPF1 phosphorylation by SMG1, and eventually mRNA degradation [63]. In principle, if an EJC-related exon–exon junction resides ≥50–55 nucleotides downstream of the termination event, then NMD initiates. Concerning the dynamics of NMD components, the binding of UPF2 with UPF1 causes a large conformational change in the UPF1 CH-domain (cysteine-histidine-rich domain), which triggers the UPF1 helicase activity. Furthermore, within the resulting decay-inducing complex (DECID), the SMG1 protein phosphorylates UPF1 that inhibits further translation termination initiation at the AUG codon [56].

UP-frameshift 1 (UPF1; Figure 1a,b), UPF2, and UPF3(a/b) proteins are the core components crucial for the NMD process in all organisms. Among these, UPF1 is primarily a cytoplasmic RNA-binding protein exhibiting RNA-dependent ATPase and RNA helicase activities for the NMD [26,27,28,29,30,31,32,33,34]. These three UPF proteins together form a ‘surveillance complex’ that triggers RNA-dependent ATPase activity of UPF1 [31,34,35,36,37,38,39,40]. Various models explaining how these NMD components recognize a PTC and recruit RNA degradation proteins have been proposed (a highly accepted model is shown in Figure 1c) [31,40,41,42,43,44,45,46,47,48,49,50,51,52,53,54,55,56]. A PTC is defined relative to the position of a downstream *cis*-acting signal, and it varies across the species [45,57,58]. The majority of models require that RNA decay be triggered once a stop codon is followed by a second signal, which specifies the stop codon as premature [31,59,60]. In mammals, the *cis*-acting signal is an exon–exon junction (EJC). For organisms or mRNAs in which EJCs do not contribute to substrate recognition in NMD, the PTC has been postulated to be distinct due to an absence of termination promoting signals from the *faux* 3′ untranslated region (UTR), particularly from poly(A)-binding protein 1 (PAB1 or PABPC1; poly(A)-binding protein cytoplasmic 1) [45,57,58,61,62,63,64]. This scheme is referred to as the ‘*faux* 3′-UTR’ model, according to which the translation termination at a normal stop codon is considered to be essentially different from translation termination at a PTC; the aberrant nature of premature termination activates the mRNA decay [31,44]. Moreover, an interaction between a terminating ribosome and a specific messenger ribonucleoprotein (mRNP) structure localized 3′ to the stop codon is required for proper termination [44,65,66,67]. The ‘*faux* 3′-UTR’ model also suggests that the proximity of the PAB1 to the PTC is essential for NMD activation [31,44,68,69].

The UPF1 protein is a key component in the NMD pathway, also termed as the master regulator. Deletion of UPF1 in yeast not only stabilizes the nonsense-containing mRNAs but also enhances nonsense codon read-through as well and inhibits degradation of prematurely terminated polypeptides [36,37,39,46]. Moreover, the work by Chan et al. [70] has shown that deletion of UPF1 induces the production of (approximately6- to 8-fold increase) novel peptide read-through. Gowravaram et al. [71] examined the UPF1 core features and identified a structural element that adopts various conformations in the nucleotide as well as RNA-bound states of UPF1. Through biochemical and single molecule assays, they have shown that a structural element modulates UPF1 catalytic activity, referred to as the ‘regulatory loop’. Interestingly, two alternatively spliced isoforms of UPF1 that differ only in length of the ‘regulatory loop’ exist in mammals [71]. UPF1 isoform_1 contains 11 aa (amino acids) insertion (353-GNEDLVIIWLR-363) in the domain 1B, which extends the ‘regulatory loop’ to 22 aa. Whereas, the UPF1 isoform_2 (the more abundant UPF1 short loop isoform) is composed of only an 11 aa ‘regulatory loop’ which weakens the UPF1 affinity for mRNA. In addition, this insertion of 11 amino acids to the ‘regulatory loop’ can considerably increase the catalytic and/or ATPase activity and result in a higher affinity for mRNA [71].

Considering such influence of the ‘regulatory loop’ in the structural dynamics of UPF1 and its control over the catalytic and/or ATPase activity, we investigated both isoforms of UPF1 in the presence or absence of the poly(U) mRNA by applying the molecular dynamics simulation (MDS) technique. Additionally, it is known that UPF1 can bind with different mRNAs efficiently [41,43,56,61,63,72,73,74], but the molecular details are still not clear. Hence, in this study, we also studied the UPF1 isoform_1 (having a longer ‘regulatory loop’) and isoform_2 binding with different mRNA motifs. The significant structural changes upon inserting the most frequently occurring cancer mutations in the UPF1 isoform_1 bound with mRNA motifs were investigated. Furthermore, preferential binding affinity of UPF1 with mRNAs in a 3’UTR length-dependent manner has been reported [17,75]. David et al. identifiedUPF1-mRNA interactions in vivo [76] and proposed that the UPF1 protein promiscuously interacts with mRNA before translation. In addition, the traced preferential binding with 3′UTRs for UPF1 at steady state originate from the selective displacement of UPF1 from coding regions by translating ribosomes [76,77]. The phosphorylated UPF1 mRNA footprint data and CLIP (cross-linking immunoprecipitation)-Seq of UPF1 in vivo identified UPF1 binding sites across transcriptome, describing UPF1 targets 3′ UTR GC-rich motifs [17,18,19,20]. Particularly, Naoto et al. [19] suggested that in 3′ UTR the GC-rich motifs (5′-CCUGGGG-3′, 5′-CCUGGGA-3′, 5′-CCUGGAA-3′, and 5′-CCUGAGA-3′) can be the target motifs in UPF1-dependent mRNA decay. Due to such specificity of UPF1 towards a particular class of mRNA motifs, and to understand the UPF1-mRNA dynamics, we performed MD simulation of UPF1 protein with differentGC-rich RNA motifs, as well as performing a comparative analysis with the AU-rich motifs (5′-UUUUUUU-3′, 5′-UUAAUUU-3′, 5′-UUAGUUU-3′, and 5′-UUGAUUU-3′) [67]. Such novel perspectives from mRNA–protein-binding pairs identified in this work may contribute to understanding the selectivity of respective partners, along with advancing NMD-associated structural dynamics and kinetics.

## 2. Results and Discussion

### 2.1. Different UPF1 Isoforms and Their Selectivity towards mRNA Motifs

**Figure 2:**
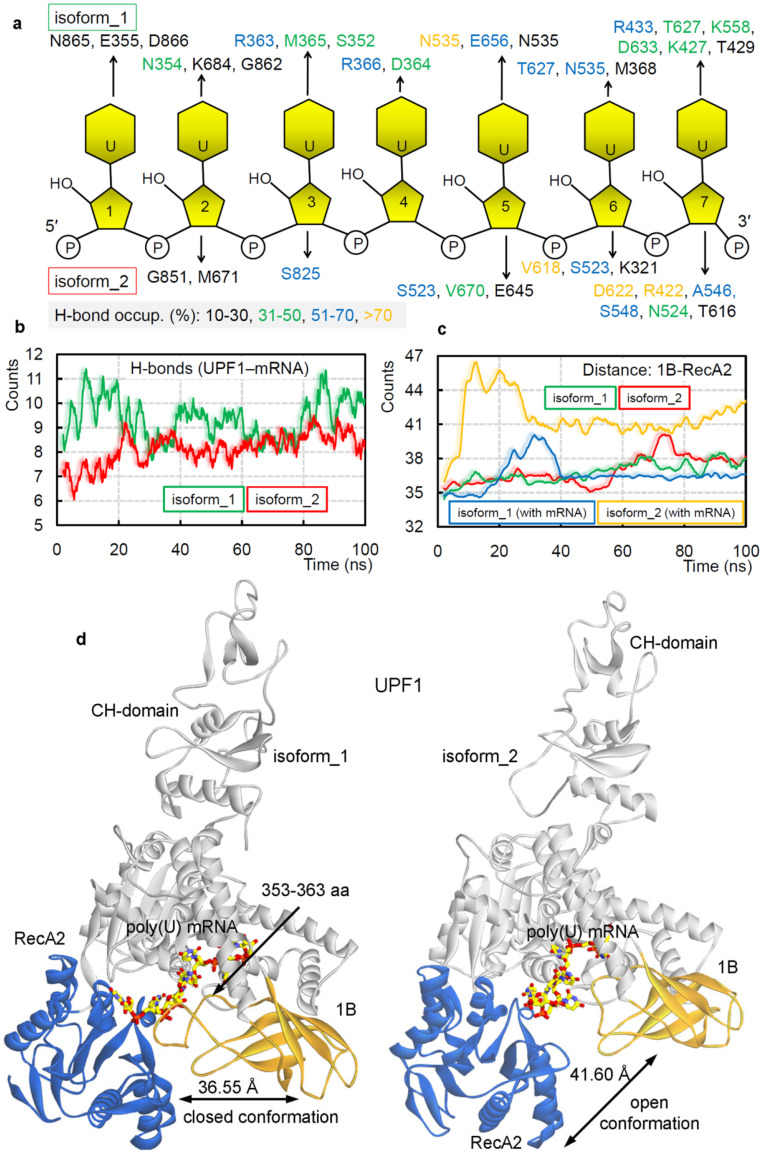
Diversity in the binding of UPF1 isoforms with poly(U) mRNA, reflected in their structural dynamics. (**a**) Individual UPF1 amino acids making long-lasting interactions with the nucleotides having occupancy ≥10%/ns. (**b**) Intermolecular hydrogen bond interactions between UPF1 isoform_1 or isoform_2 with the poly(U) mRNA motifs. (**c**) The distance center of mass between 1B and RecA2 domains from both isoforms in the presence/absence of the poly(U) mRNA. (**d**) The structural dynamics of the ‘regulatory loop’ in presence (isoform_1) and absence of (isoform_2) of the 11 aa insertion (353-GNEDLVIIWLR-363). The averaged UPF1-mRNA coordinates extracted from 100 ns (nanosecond) MD (molecular dynamics) simulations, with labeled average distance marked based on data from panel (**c**).

In mammals, two alternatively spliced isoforms for theUPF1 protein were identified that differed in length of the ‘regulatory loop’ from 1B domain, positioned at the mRNA binding interface. Such insertion of 11 aa (353-GNEDLVIIWLR-363) in the ‘regulatory loop’ made the UPF1 isoform_1 capable of a 2-fold increase in UPF1 translocation and ATPase activities [71] compared withUPF1 isoform_2. Applying the MDS approach, we identified the selectivity of amino acids from two different UPF1 isoforms with poly(U) mRNA motifs (Figure 2a,b). Binding interfaces and the number of protein–mRNA interactions (hydrogen bond; H-bond) theorized that UPF1 isoform_1 would havea higher number of interactions with poly(U) mRNA compared with that of the UPF1 isoform_2 (Figure 2b). We further traced the residues resulting instable hydrogen bonding with an individual mRNA (occupancy ≥ 10 %/ns; Figure 2a). The UPF1 isoform_1 was found forming interactions with each nucleotide of poly(U) mRNA, whereas the isoform_2 lacked such a binding pattern (Figure 2a). Apart from several isoform-specific UPF1-mRNA interactions (Figure 2a), the following amino acids (isoform_2/isoform_1) were common making interactions with mRNAs from both UPF1 isoforms: G851/G862, E645/E656, D622/D633, R422/R433, N524/N535, and T616/T627 (Figure 2a). Moreover, the presence of 11 aa insertion in the ‘regulatory loop’ for the UPF1 isoform_1 formed an interface for several residues to form H-bond interactions with the mRNA motifs—particularly the E355, N354, and R363 residues were involved in such binding (Figure 2a).

The dynamics of different UPF1 isoforms over MD simulation (100 ns) time, demonstrates that the 11 aa insertion in the ‘regulatory loop’ induces movements between 1B and RecA2 domains. Considering such conformational switch, we computed the distance center of mass between 1B and RecA2 domains (Figure 2c). The findings highlight that in the absence of the insertion, UPF1 isoform_2 (with mRNA) has the highest distance, compared with that in theUPF1 isoform_1 (Figure 2c). On the contrary, the apo-form (UPF1 isoform_1 or isoform_2) systems lacked any such distinctive behavior (Figure 2c). These data suggest that intact binding between 1B and RecA2 domains in the presence of 11 aa insertion (isoform_1) within the ‘regulatory loop’ may be responsible for the induced catalytic and/or ATPase activity, as observed by Gowravaram et al. [71]. The averaged UPF1–mRNA coordinates extracted from 100 ns (nanosecond) MD simulations (Figure 2d) defines a ‘closed conformation (for isoform_1)’ and ‘open conformation (for isoform_2)’ between the 1B and RecA2 domains. In addition, similar conformational dynamics have been previously reported for one of the ATPases: DEAH-box adenosine triphosphatases by Florian et al. [73], their crystal structures [73] revealed that the RecA2 domain in presence of RNA formed an ‘open conformation’ and that the apo-form resembled a ‘closed conformation’. However, such two distinct conformations for the UPF1 protein were observed in our analysis having poly(U) mRNA in the system and in the presence or absence of the insertion in the ‘regulatory loop’ (Videos S1–S2).

### 2.2. GC- and AU-Rich mRNA Motifs Binding Pattern with UPF1 Isoforms

PTC-containing mRNAs are rapidly degraded via the NMD pathway (Figure 1), and it has been proposed that UPF1 (the master regulator) is often associated with these mRNAs [74,78]. In addition, the CLIP-Seq studies suggest that UPF1 binds mRNA rather non-specifically, but tends to accumulate in GC-rich motifs of 3′ UTRs [17,18,19]. Therefore, to investigate the binding pattern and selectivity of UPF1 with different mRNA motifs, we performed MD simulations on different GC-rich mRNA motifs with the UPF1protein, as well as made a comparative analysis with AU-rich sequences. For constructing different GC- and AU-rich mRNA motifs, model structures for the MD simulations with UPF1, the poly(U) mRNA (5′-UUUUUUU-3′) from the X-ray structure with UPF1 (pdb id: 2xzo [37]), was considered as the template. These eight investigated mRNA motifs (GC- (5′-CCUGGGG-3′, 5′-CCUGGGA-3′, 5′-CCUGGAA-3′, 5′-CCUGAGA-3′) and AU-rich motifs (5′-UUUUUUU-3′, 5′-UUAAUUU-3′, 5′-UUAGUUU-3′, 5′-UUGAUUU-3′), by our MD simulation approach, can be some of the many mRNAs with whichUPF1 can interact.

**Figure 3:**
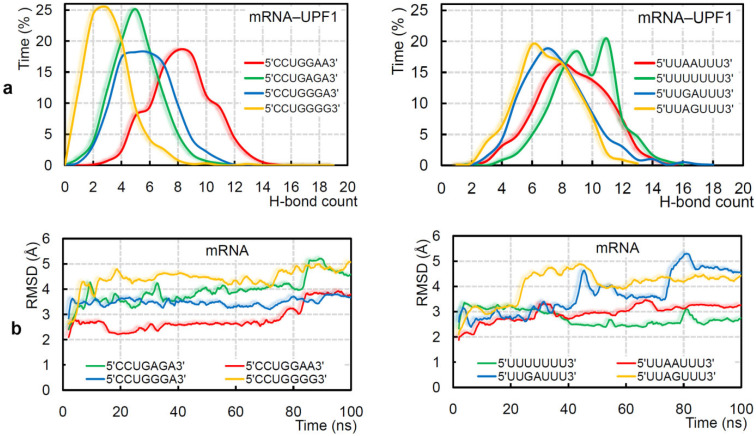
Adopted binding patterns by UPF1 (isoform_1) with different mRNA motifs. (**a**) Frequency of the number of hydrogen bond interactions formed between UPF1 (isoform_1) and different GC-rich (5′-CCUGGGG-3′, 5′-CCUGGGA-3′, 5′-CCUGGAA-3′, and 5′-CCUGAGA-3′) or AU-rich (5′-UUUUUUU-3′, 5′-UUAAUUU-3′, 5′-UUAGUUU-3′, and 5′-UUGAUUU-3′) motifs. (**b**) Root-mean-square deviation (RMSD) of atomic positions (excluding hydrogen atoms) for the GC- and AU-rich mRNA motifs computed in the presence of the UPF1 protein.

The intermolecular interactions of different protein–mRNA systems revealed that different mRNA motifs vary in binding affinity with UPF1 (isoform_1; Figure 3a). Explicitly, the 5′-CCUGGGG-3′, 5′-CCUGGGA-3′, 5′-CCUGGAA-3′, and 5′-CCUGAGA-3′mRNA motifs that represent 246 UPF1 targets [19] individually have slightly different affinity with the UPF1 isoform_1 (Figure 3a; left panel). Particularly, 5′-CCUGAGA-3′ and 5′-CCUGGAA-3′ have higher interactions with the UPF1 protein compared with other GC-rich motifs, whereas 5′-CCUGGGG-3′ has the lowest protein–mRNA interactions (Figure 3a; left panel). Among the AU-rich RNA motifs, the 5′-UUAAUUU-3′, 5′-UUAGUUU-3′, and 5′-UUGAUUU-3′ sequences represented a reduced binding with the UPF1 compared with that of the poly(U) mRNA (Figure 3a; right panel). The 5′-UUAGUUU-3′ motif has the least binding with UPF1 among AU-rich mRNA motifs (Figure 3a; right panel). The binding affinity of GC- and AU-rich mRNA motifs with UPF1 suggest that comparatively the GC-rich mRNA motifs have better binding in the majority of cases (Figure 3a). A corresponding trend of difference in their RMSDs (root-mean-square deviation of atomic positions) was traced, i.e., the 5′-CCUGGAA-3′ and poly(U) mRNA motifs were found to be highly stable throughout the MD simulations (Figure 3b). In addition, the 5′-CCUGGGG-3′ and 5′-UUGAUUU-3′ motifs with the least binding with UPF1 isoform_1 have higher flexibility (Figure 3b). Overall, the GC-rich motifs in the presence of UPF1 were found to show less fluctuation (more stable) in structure compared with that of the AU-rich motifs (isoform_1; Figure 3b).

**Figure 4:**
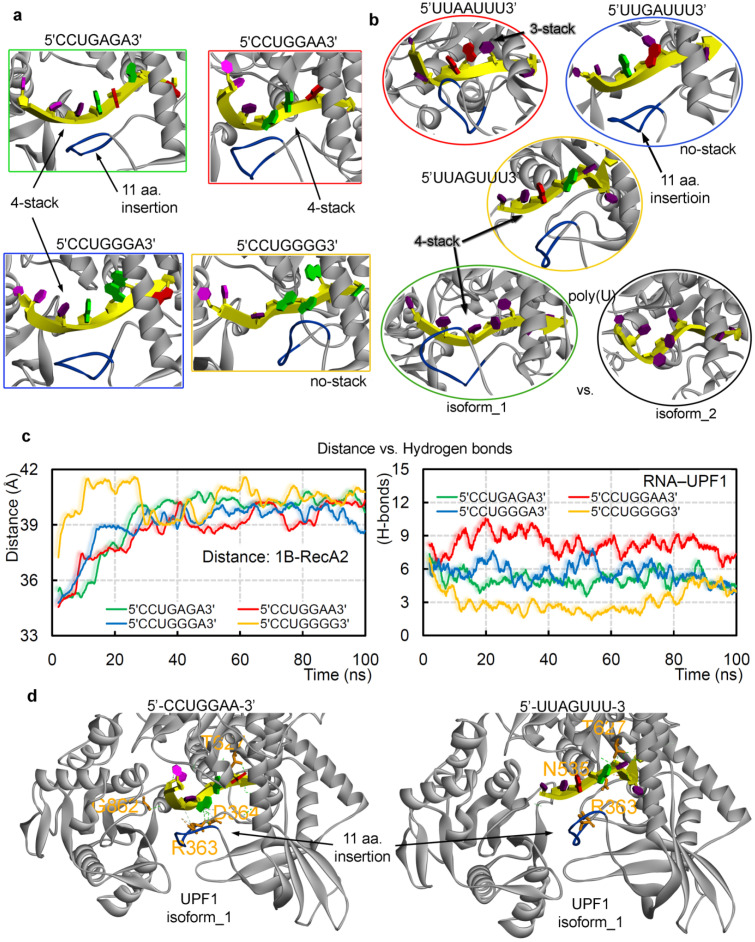
The dynamics of different UPF1-mRNA simulated systems. The (**a**,**b**) stacking pattern interactions formed by different GC- and AU-rich mRNA motifs with UPF1 isoforms and averaged structures retrieved from the 100 ns MD simulations are presented. For UPF1 isoform_1, the insertion 353-GNEDLVIIWLR-363 is marked in blue color. (**c**) The distance center of mass computed between 1B and RecA2 domains of UPF1 isoform_1 with GC-rich mRNA motifs (left panel), as well as the UPF1-mRNA intermolecular hydrogen bond interactions (right panel). (**d**) UPF1 isoform_1 residues that were found common, as well as those making high occupancy (≥50 ns) interactions with GC-rich motifs (R363, D364, T627, and G862) or AU-rich motifs (R363, N535, and T627). As an example, the 5′-UUAGUUU-3′ and 5′-CCUGGAA-3′ motifs are represented with the UPF1 protein. Nucleotide color scheme; adenine (A) in red color, uracil (U) in dark purple, cytosine (C) in light purple, and guanine (G) in green.

Over the MD simulation time, it was observed that the poly(U) mRNA motif forms a 4-stack pattern when making interactions with UPF1 isoform_1 (Figure 4a,b), whereas the mRNA motif lacks such conformation in the presence of UPF1 isoform_2 (Figure 4b, bottom panel). These differences in the binding pattern of mRNA with two isoforms of UPF1 may explain the cause of induced catalytic activity for the UPF1 isoform_1 [71]. Particularly, among the GC-rich motifs, except 5′-CCUGGGG-3′, all other studied mRNA motifs formed a 4-stack pattern of binding with UPF1 (Figure 4a). Among the AU-rich motifs, the 5′-UUAAUUU-3′ and 5′-UUAGUUU-3′ formed a 3-stack and 4-stack pattern, respectively, whereas 5′-UUGAUUU-3′ lacked such formation (Figure 4b). Correlating the distance center of mass between 1B and RecA2 domains (Figure 4c; left panel) with the mRNA–UPF1 interactions (Figure 4c; right panel) suggests that as the distance between both domains increases (open conformation), the hydrogen bonds between mRNA–UPF1 (isoform_1) decreases (Figure 4c). For example, the system with 5′-CCUGGAA-3′ motif has less distance between 1B and RecA2 domains (closed conformation) and higher mRNA–UPF1 interactions, whereas a contrary behavior is observed for the 5′-CCUGGGG-3′ motif (isoform_1; open conformation; Figure 4c). Furthermore, we studied GC-rich mRNA motifs with UPF1 isoform_2, which is found to be abundant in cells, and compared findings with the UPF1 isoform_1 (Figures S2 and S3). The RMSD of individual GC-rich mRNA motifs with the both UPF1 isoforms, suggests that the same mRNA motifs behave differently when bound with isoform_1 or isoform_2 (Figure S2)—i.e., mRNA motifs with UPF1 isoform_2 were found to be highly flexible when compared with that of the UPF1 isoform_1. The 5′-CCUGGGG-3′ motif showed the least binding with UPF1 isoform_2, similar as with the UPF1 isoform_1 (Figure S2). These weak-binding mRNA motifs with the UPF1 isoform_2 resulted in an increased distance between 1B and RecA2 domains (open conformation; Figure S2).

Additionally, UPF1 isoform_1 with the 5′-UUGAUUU-3′ motif formed an ‘open conformation’ between 1B-RecA2 domains, whereas the other two AU-rich systems (5′-UUAAUUU-3′ and 5′-UUAGUUU-3′) showed a ‘closed conformation’ (similar to when complexed with the poly(U) mRNA motif). These ‘open conformations’ of the UPF1 (isoform_1) protein in the presence of the 5′-CCUGGGG-3′ and 5′-UUGAUUU-3′ mRNA motifs correlated with the conformation dynamics of UPF1 isoform_2 which is suggested to have comparatively less catalytic activity [71]. Our findings propose that to induce the catalytic activity of the UPF1 helicase, a ‘closed conformation’ between 1B and RecA2 domains, as well as a specific stacking pattern in the mRNA transcript may be needed (Figure 4). Furthermore, tracing the interacting residues of the UPF1 protein with different mRNA motifs (Tables S1 and S2) demonstrated that a few amino acids were found to be common, resulting in a high occupancy (≥50 ns; Figure 4d) binding with GC-rich motifs (R363, D364, T627, and G862) or AU-rich motifs (R363, N535, and T627).

### 2.3. UPF1_A839T_ and UPF1_P533L/T_ Cancer Mutants Influencing the UPF1-mRNA Binding

**Figure 5:**
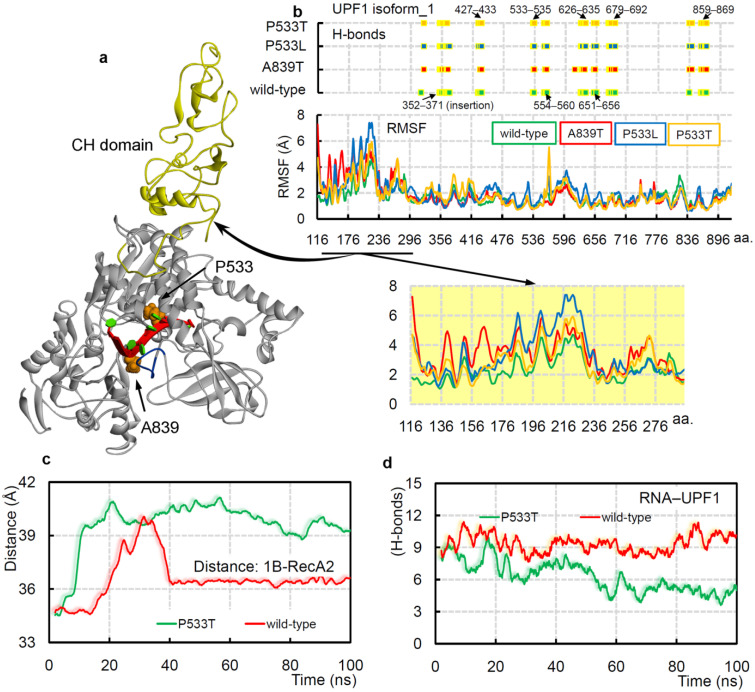
The cancer variants UPF1_A839T_ and UPF1_P533L/T_ can influence the protein–mRNA binding dynamics. (**a**) UPF1 isoform_1 structure representing mutated residues in its RNA-binding pocket, and the CH domain is colored in yellow. The 11 aa insertion (353-GNEDLVIIWLR-363) for UPF1 isoform_1 is also highlighted in blue. (**b**) The protein–poly(U) mRNA intermolecular hydrogen bond interactions in wild-type and different mutated systems. Amino acids from the following range of the UPF1 isoform_1 protein were involved in poly(U) mRNA binding: S352–D371, K427–R433, P533–N535, R554–R560, C626–R635, Q651–E656, P679–S692, S836–Q841, and Q859–R869. The bottom panel represents RMSFs (root-mean-square fluctuations) of individual amino acids of UPF1 (isoform_1) under different conditions. The regions formed by the CH-domain showing high RMSF fluctuations are highlighted. (**c**) The distance center of mass between 1B and RecA2 domains of UPF1 isoform_1 with poly(U) mRNA in the UPF1_P533T_mutated and wild-type systems. (**d**) The UPF1–poly(U) mRNA intermolecular interactions from the UPF1_P533T_ and wild-type systems.

Point mutation often alters the normal functioning of proteins. Therefore, to investigate such an effect on the UPF1 (isoform_1) helicase activity, we performed MD simulations upon inserting the commonly occurring UPF1 cancer variants in the presence of poly(U) mRNA—A839T and P533L/T (cBioPortal [79])—positioned at the mRNA-binding interface. Upon inserting mutations in the UPF1 protein, a reduction in the protein–mRNA bindings was observed, and such significant changes were traced for the UPF1_P533T_ system. The change in binding motifs of UPF1 upon mutating with poly(U) mRNA was identified, and amino acids from the insertion (353-GNEDLVIIWLR-363; isoform_1) in the ‘regulatory loop’ were found making stable interactions with the mRNA motifs (Figure 5a,b). Interestingly, the mutated UPF1_P533L/T_ residue itself was found interacting with mRNA in the mutated as well as the wild-type systems, whereas the protein with mutated residue A839T lacked such behavior with the mRNA motif. Particularly, the UPF1_A839T_ system had a distinctive interaction with mRNA compared with other simulated systems (Figure 5b). For example, the residue E614 formed stable (highest occupancy) interactions with mRNA in UPF1_A839T_, whereas other all simulated systems lacked such binding (Figure 5b). Moreover, residues Y316, Q369, and T429 were binding with mRNA only in the wild-type systems, and similarly, residues K321, G353, G630, G681, and Q841 formed interactions only in the mutated systems (Figure 5b).

Overall, the RMSF (root-mean-square fluctuations) findings suggest that the UPF1 (isoform_1) amino acids have higher flexibility in the mutated systems (Figure 5b, bottom panel) compared with that of the wild-type form. In addition, significant RMSF fluctuation differences were observed in the CH domain (cysteine-histidine-rich domain), which could be an allosteric effect upon inserting mutations in the RNA-binding sites (Figure 5b). The conformational dynamics of protein upon inserting cancer variants in the presence or absence of mRNA suggest that the UPF1_P533T_ system in both conditions constitute an ‘open conformation’ for the 1B and RecA2 domains, whereas the UPF1_P533L_ and UPF1_A839T_ lack such domain displacements. Moreover, in the UPF1_P533T_ system, the mRNA does not form a stable stacking pattern, as is observed in the wild-type system (isoform_1). Comparing the distance center of mass between the 1B and RecA2 domains with the intermolecular UPF1-mRNA interactions revealed that as the distance between domains gradually increases, the protein–mRNA interactions decreased (Figure 5c,d, and Video S3). These data could support the hypothesis that the ‘open conformation’ between 1B and RecA2 domains may reduce the catalytic activity, whereas the ‘closed conformation’ can induce such activity.

## 3. Materials and Methods

### Systems Build-Up for Different mRNA Motifs with UPF1

Different structures of UPF1 with ATP or mRNA are available in the Protein Data Bank database (pdb; http://www.rcsb.org/pdb; accessed on March 2021); however, the majority of them lack the CH domain, which is crucial for the proper functioning of the UPF1 protein. Therefore, in this study, we used the complete optimized UPF1structure (pdb id: 2wjv [67]) retrieved from our previous study [40]. The UPF1 (pdb id: 2wjv [67]) is an isoform_2 crystal structure having 11 aa less (353-GNEDLVIIWLR-363) in the ‘regulatory loop’ compared with the isoform_1 (http://www.rcsb.org/pdb; accessed on March 2021). The UPF1 isoform_1 structure was modeled by considering the isoform_2 crystal structure as the template (pdb id: 2wjv [67]; Figure 1a,b). The missing amino acids in the tertiary structures of UPF1 in both isoforms were built using the Swiss model, as well as using the ‘prepare protein’ protocol implemented in the macromolecules module of the BIOVIA Discovery Studio Client v18.1 (Dassault Systèmes, BIOVIA Corp., San Diego, CA, USA) program [41,80]. Superimposing both UPF1 isoform modeled structures (build based on the pdb id: 2wjv [67]) with different structures available (UPF1 isoform_2 (pdb id: 2xzp [37]) and UPF1_isoform_1 (pdb id: 6ej5 [71])) suggests a strong correlation between conformations (Figure S1). The UPF1 crystal structure containing the CH domain (pdb id: 2wjv [67]) lacks the mRNA, and therefore, the position of the poly(U) mRNA motif was used from the pdb id: 2xzo X-ray structure (UPF1 without the CH domain) [37]. Using the ‘superimpose module’ in BIOVIA Discovery Studio v18.1 package, the position of poly(U) mRNA with both UPF1 isoforms was defined. The minimization of the modeled UPF1–poly(U) mRNA systems were performed using the ‘smart minimizer’ algorithm and applying the CHARMm (Chemistry at Harvard Macromolecular Mechanics) forcefield [81], with other parameters set as default in the BIOVIA Discovery Studio v18.1 package.

Systems for the MD simulations of UPF1 with GC-rich (5′-CCUGGGG-3′, 5′-CCUGGGA-3′, 5′-CCUGGAA-3′, and 5′-CCUGAGA-3′) and AU-rich (5′-UUUUUUU-3′, 5′-UUAAUUU-3′, 5′-UUAGUUU-3′, and 5′-UUGAUUU-3′) mRNA motifs were generated using the Molecular Operating Environment (MOE; Chemical Computing Group Inc., Montreal, QC, Canada) package. A similar position as that of the poly(U) mRNA motif with UPF1 from the X-ray structure (pdb id: 2xzo) [37] was used for these GC/AU-rich mRNA–UPF1 systems. Subsequently, the UPF1 protein with different mRNA motifs were optimized in the MOE package applying the CHARMM27 forcefield [82]. The cancer mutations retrieved from the cancer genomics database cBioPortal [79] were inserted in the UPF1 structure using the MOE modules. Tertiary coordinates of mRNA as input parameters for the GROMACS 4.6.5 package [83] were retrieved using the CHARMM-GUI server [84]. Applying CHARMM27 forcefield, the following 21 different protein–RNA systems were simulated using GROMACS 4.6.5 [85]: UPF1 isoform_1 apo-form and with poly(U) mRNA; UPF1 isoform_2 apo-form and with poly(U) mRNA; UPF1 isoform_1 and isoform_2 with GC-rich mRNA motifs (5′-CCUGGGG-3′, 5′-CCUGGGA-3′, 5′-CCUGGAA-3′, and 5′-CCUGAGA-3′); UPF1 isoform_1 with AU-rich mRNA motifs (5′-UUAAUUU-3′, 5′-UUAGUUU-3′, and 5′-UUGAUUU-3′) and the mutated UPF1 isoform_1 (A839T, P533L, or P533T) apo-form and with poly(U) mRNA. These protein–mRNA systems were simulated in a 10 Å thick dodecahedron box and solvated using simple point charge (SPC) water molecules [86]. Net charge of the individual system was neutralized by adding the appropriate number of Na^+^ and Cl^−^ counter ions in the MD simulation box. Periodic boundary conditions were employed in all three dimensions, and to relax possible steric crashes, the energy of each system was minimized for 50,000 steps of the steepest descent algorithm. Different electrostatic interactions were treated using the particle mesh Ewald (PME) method [87], and the LINCS (linear constraint solver) algorithm [88] was used to constrain the bond lengths for each simulated system. In addition, the cutoff distance for van der Waals and Coulomb interactions were set to 10 Å. Subsequently, all modeled systems were equilibrated in an NpT-ensemble (isobaric–isothermal) simulation for 1000 ps. The temperature and pressure were maintained at 300 K and 1 bar using V-rescale thermostat [88] and Parrinello–Rahman barostat [89], respectively. A leapfrog integrator [90] was used to propagate the dynamics of each system. The MDS production run was carried out for 100 ns, and the trajectories/coordinates were saved every 10 ps (picosecond), which were analyzed using GROMACS tools. Hydrogen bonds (H-bonds) were calculated using a donor-acceptor atom cutoff distance of 3.5 Å and intermolecular donor-H-acceptor angle cutoff ≥160°–180°. The VMD (Visual Molecular Dynamics) tool [91] was used to visualize the MD simulated trajectories, and an MOE/BIOVIA Discovery Studio visualizer was used for the protein–mRNA structure representation.

## 4. Conclusions

In this work, we investigated two alternatively-spliced UPF1 isoforms with different mRNA motifs, and we traced significant structural dynamics by inserting cancer-derived mutations in the UPF1 mRNA binding pocket. To this end, we suggest that 11 aa insertion (353-GNEDLVIIWLR-363) in the ‘regulatory loop’ could be responsible for the ‘open (isoform_2)’ and ‘closed (isoform_1)’ conformations between 1B and RecA2 domains, which may impact the catalytic activity of the UPF1 helicase. These two distinct conformations of UPF1 correspond to the important stacking pattern observed in the mRNA motifs, i.e., the absence of a stacking formation in isoform_2 results in an ‘open conformation’. The amino acids E355, N354, and R363 from the insertion region for UPF1 isoform_1 induced interactions with the mRNA motif.

Binding affinities of GC- and AU-rich mRNA motifs with UPF1 highlighted that GC-rich mRNAs have better binding with the protein in the majority of studied cases. In addition, when complexed with the UPF1 protein, the GC-rich motifs have lower fluctuations in their structure compared with that of the AU-rich motifs. Except for 5′-CCUGGGG-3′, all other simulated GC-rich mRNA motifs formed a 4-stack binding pattern with the UPF1 protein. Additionally, it was observed that as the distance between 1B-RecA2 domain increases forming an ‘open conformation’, it has a decline in the UPF1-mRNA intermolecular interactions. For example, the system with 5′-CCUGGAA-3′ motif had less distance between 1B-RecA2 domains (closed conformation) and higher mRNA–UPF1 interactions, whereas contrary behavior was observed for the 5′-CCUGGGG-3′ (open conformation) sequence. Among the AU-rich motifs, the poly(U) mRNA had the highest binding affinity with the UPF1 and that with least binding affinity was the 5′-UUAGUUU-3′ motif. Particularly, 5′-UUAAUUU-3′ and 5′-UUAGUUU-3′ formed a 3-stack and 4-stack pattern, respectively, whereas 5′-UUGAUUU-3′ lacked such formation, resulting in an ‘open conformation’ between 1B-RecA2 domains.

A few high occupancy R363, D364, T627, and G862 residues from UPF1 isoform_1 were common binders among the GC-rich motifs, as were residues R363, N535, and T627 among the AU-rich motifs. Comparing different GC-rich mRNA motifs binding with both UPF1 isoforms highlighted that mRNA motifs behave slightly differently when bound with either of the isoforms, and particularly, mRNA motifs with UPF1 isoform_2 were more flexible. The amino acid positioned at 533 in the UPF1 isoform_1 protein was found interacting with mRNA in the mutated (UPF1_P533L/T_) as well as in the wild-type systems, whereas the UPF1_A839T_ systems have distinctive interactions with the mRNA motif. Particularly, in the cancer-associated mutated systems, the mRNA motif with UPF1_P533T_ lacks the stacking interactions, due to which the distance between 1B-RecA2 domains increased and the intermolecular protein–mRNA interactions showed a declining trend. From these data it could be proposed that the increased affinity betweenUPF1-mRNA components, should contribute to the enhanced RNA-dependent ATPase/helicase activity of the UPF1 protein that is necessary for the NMD pathway. These novel perspectives from identified mRNA–UPF1 binding pairs can contribute to understanding the selectivity of respective partners, as well as advancing NMD-associated dynamics and kinetics.

## Data Availability

Data is contained within the article or supplementary material.

## Supplementary Materials

The following are available online at https://www.mdpi.com/article/10.3390/ijms222312744/s1.

## Abbreviations

aa	amino acids
CLIP	cross-linking immunoprecipitation
CH domain	cysteine-histidine-rich domain
CHARMm	Chemistry at Harvard Macromolecular Mechanics
DECID	decay-inducing complex
EJC	exon–exon junction complex
eRF1	eukaryotic translation termination factor 1
H-bond	hydrogen bond
LINCS	linear constraint solver
mRNP	messenger ribonucleoprotein
MDS	molecular dynamics simulation
MOE	Molecular Operating Environment
NGD	no-go decay
NMD	nonsense-mediated mRNA decay
NSD	non-stop decay
ns	nanosecond
PAB1	poly(A)-binding protein cytoplasmic 1
pdb	protein data bank
PME	particle mesh Ewald
PTC	premature termination codon
ps	picosecond
RMSD	root-mean-square deviation
RMSF	root-mean-square fluctuations
SMG	suppressors with morphogenetic effect on genitalia
SPC	simple point charge
uORF	upstream open reading frames
UPF1	UP-frameshift 1
UTR	untranslated region
VMD	Visual Molecular Dynamics
