# Supplementary material for: Molecular Determinants and Specificity of mRNA with Alternatively-Spliced UPF1 Isoforms, Influenced by an Insertion in the ‘Regulatory Loop’
Source: Int J Mol Sci. 2021 Nov 25;22(23):12744. doi: 10.3390/ijms222312744 (PMC8657986; doi:10.3390/ijms222312744)
Supplement: Supplementary file 1 [file ijms-22-12744-s001.zip › Kalathiya_Supp-Mat-R2.pdf]

Molecular determinants and specificity of mRNA with alternatively-spliced UPF1 isoforms, influenced by an insertion in the 'regulatory loop'

Monikaben Padariya[1], Robin Fahraeus[1,2,3,4], Ted Hupp[1,5]* and Umesh Kalathiya[1]*

[1]International Centre for Cancer Vaccine Science, University of Gdansk, ul. Kładki 24, 80-822 Gdansk, Poland
[2]Inserm UMRS1131, Institut de Génétique Moléculaire, Université Paris 7, Hôpital St. Louis, F-75010 Paris, France
[3]Department of Medical Biosciences, Building 6M, Umeå University, 901 85 Umeå, Sweden
[4]RECAMO, Masaryk Memorial Cancer Institute, Zlutykopec 7, 65653 Brno, Czech Republic
[5]Institute of Genetics and Cancer, University of Edinburgh, Edinburgh, Scotland EH4 2XR, UK
*Correspondence: ted.hupp@ed.ac.uk (T.H), umesh.kalathiya@ug.edu.pl (U.K)

Supporting Materials

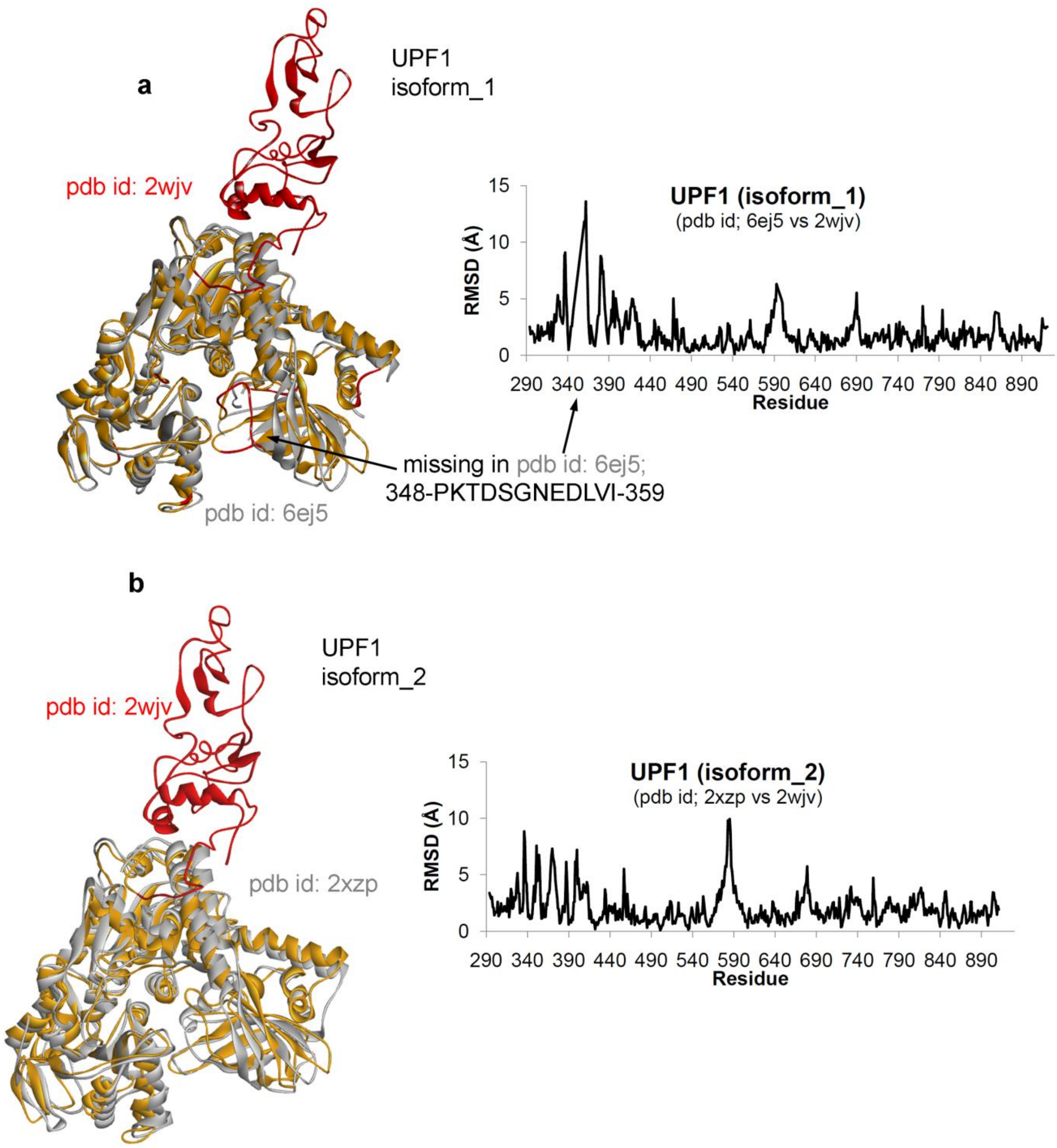

Figure S1. The modeled isoform structures of the UPF1 protein investigated in this study, superimposed with different available structures in the Protein Data Bank database (http://www.rcsb.org/pdb). **(a)** UPF1 isoform_1 modeled structures (pdb id: 2wjv) compared with pdb id. 6ej5, the missing residues in the structure pdb id. 6ej5 and are available in our modeled structure (pdb id: 2wjv) are marked red. Right panel represents the RMSD computed from both structures when superimposing over each other. Higher flexibility in the initial region is seen due to missing 'regulatory loop' residues 348-PKTDSGNEDLVI-359 in the structure; pdb id. 6ej5. (**b)** UPF1 isoform_2 modeled structures (pdb id. 2wjv) vs. (pdb id. 2xzp), the missing CH-

domain in the pdb id: 2xzp is marked in red color. Right panel represents the RMSD computed from both structures, when superimposing each other.

Table S1. Different GC-rich mRNA motifs make long lasting hydrogen bond interactions with the UPF1 isoform_1 protein during 100 ns MD simulations. The H-bond interactions with occupancy ≥ 10% are presented in this table.

**5'CCUGAGA3'**			**5'CCUGGAA3'**			**5'CCUGGGA3'**			**5CCUGGGG3'**		
**UPF1**	**mRNA**	**Occup. (%)**	**UPF1**	**mRNA**	**Occup. (%)**	**UPF1**	**mRNA**	**Occup. (%)**	**UPF1**	**mRNA**	**Occup. (%)**
Gly862	C1	68.06	Asn535	A5	76.15	Asp364	G4	47.70	Val629	G5	76.15
Asp364	A4	62.18	Arg363	G3	70.76	Arg366	A6	41.52	Arg366	G6	38.82
Arg363	U2	56.19	Met365	G4	58.78	Arg363	G3	39.42	Asn865	C1	34.13
Thr627	A6	51.30	Gly862	U2	56.89	Arg366	G4	37.92	Glu313	G3	16.77
Lys321	A6	26.85	Asn535	A6	51.80	Met365	G4	31.24	Ser559	G6	13.37
Asp317	A6	22.55	Thr627	A6	48.30	Asp371	A6	30.64	Arg869	U2	11.98
Glu656	G5	21.76	Asn535	A5	46.31	Val681	G3	25.25	Thr429	G6	10.38
Ile359	U2	17.96	Asp364	G4	40.42	Gln651	G5	21.06			
Ser559	A6	16.37	Arg869	C1	38.52	Ser836	G3	19.86			
Asn535	A6	14.47	Arg366	A6	38.02	Lys684	C1	19.06			
Ser534	A6	12.77	Ser430	A5	29.54	Gly862	U2	18.76			
Arg366	G5	12.57	Ser534	G4	28.94	Glu656	G5	18.66			
			Pro679	C1	26.45	Asn865	C0	18.26			
			Arg366	A6	20.56	Ser559	A6	17.76			
			Arg560	A6	17.17	Gly862	C1	17.66			
			Ser836	G3	16.77	Asp866	C0	17.66			
			Arg366	A5	15.77	Arg363	G4	15.47			
			Asn354	G3	15.57	Val681	U2	12.18			
			Ser559	A6	12.77	Asn535	G5	12.18			
			Arg868	C1	12.18	Asn865	C0	10.98			
			Ser352	G3	11.38	Asn535	A6	10.88			
			Val680	G3	10.78%						

Table S2. Different AU-rich mRNA motifs form a long lasting hydrogen bond interactions with the UPF1 isoform_1 protein during 100 ns MD simulation. The H-bond interactions with occupancy ≥ 10% are presented in this table.

5'UUUUUUU3'			5'UUAAUUU3'			5'UUGAUUU3'			5'UUAGUUU3'		
**UPF1**	**mRNA**	**Occup. (%)**	**UPF1**	**mRNA**	**Occu p. (%)**	**UPF1**	**mRNA**	**Occup. (%)**	**UPF1**	**mRNA**	**Occup. (%)**
Asn535	U5	70.46	Ile359	A3	76.25	Asn535	U6	76.05	Thr627	U6	64.17
Arg363	U3	68.16	Asn535	U6	52.50	Arg363	A4	53.49	Ala557	U7	55.79
Thr627	U6	66.57	Thr627	U6	51.50	Asp364	U5	51.50	Gly862	U2	42.81
Asn535	U6	56.29	Asp371	U6	49.10	Ser559	U7	43.81	Arg554	U7	39.82
Arg366	U4	54.29	Arg433	U7	46.61	Asn535	U6	40.62	Glu313	G4	38.72
Glu656	U5	51.60	Arg869	A3	46.31	Ala557	U7	37.92	Thr627	U7	38.32
Arg433	U7	50.40	Asn535	U5	46.01	Cys625	U7	36.53	Thr627	U7	35.33
Met365	U3	48.50	Leu357	U2	45.61	Leu357	G3	34.13	Asp371	U6	23.65
Asn354	U2	45.41	Glu355	U2	40.02	Thr627	U6	33.53	Asn535	U6	23.35
Thr627	U7	43.51	Met368	U6	36.73	Met365	U5	30.54	Arg869	U2	21.86
Ser352	U3	43.31	Met365	U5	32.73	Tyr316	G3	25.55	Ser534	U6	21.36
Asp364	U4	40.22	Gly630	U7	32.24	Asn535	U7	22.26	Ser430	U5	18.86
Lys558	U7	37.23	Val681	U1	26.15	Arg554	U7	21.56	Ser534	U5	17.86
Asp633	U7	36.43	Asn535	U5	23.35	Gly630	U7	20.06	Ser559	U6	16.97
Lys427	U7	35.93	Asp633	U7	21.16	Gln651	U5	19.46	Ser836	A3	12.97
Asn535	U5	24.05	Arg869	U2	21.06	Glu656	U5	17.17	Arg363	G4	11.98
Asn865	U1	21.06	Gly862	U2	18.56	Leu555	U7	13.47	Arg366	U5	11.28
Thr429	U7	17.37	Cys625	U7	17.37	Val680	U5	12.97			
Glu355	U1	14.87	Asp866	U1	14.47	Ile359	G3	12.48			
Lys684	U2	12.67	Arg366	U5	14.47	Asn354	G3	12.38			
Asp866	U1	12.57	Gln651	A4	14.27	Ser430	U6	11.88			
Met368	U6	12.48	Ser430	U5	12.77						
Gly862	U2	10.48	Glu313	A4	11.98						
			Ser534	A4	11.38						

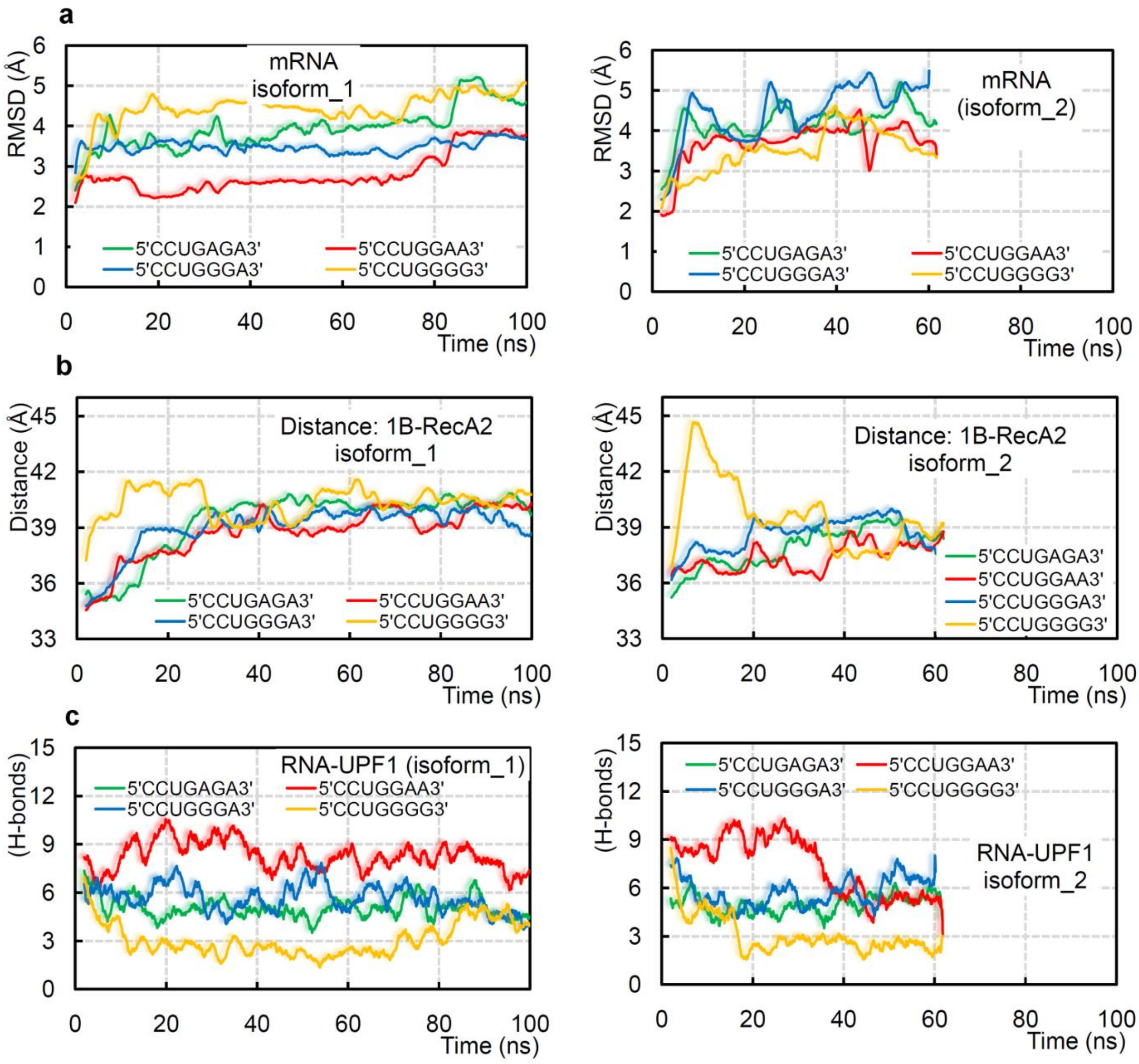

Figure S2. Both isoforms of UPF1 investigated in the presence of GC-rich mRNA motifs (5’-CCUGGGG-3’, 5’-CCUGGGA-3’, 5’-CCUGGAA-3’, and 5’-CCUGAGA-3’). **(a)** Root-mean-square deviation (RMSD) of atomic positions (excluding hydrogen atoms) for GC-rich mRNA motifs in the presence of UPF1. **(b)** The distance centre of mass computed between 1B and RecA2 domains of UPF1 isoform_1 when complexed with a GC-rich mRNA motif system. **(c)** The UPF1-mRNA intermolecular hydrogen bond interactions. For UPF1 isoform_1, the MD simulation was performed for 100 ns and for UPF1 isoform_2 the MD simulation was performed for ~60 ns. During this MDS time frame, it was observed that the UPF1 protein system has reached the equilibrium state in both UPF1 isoforms (Figure S3).

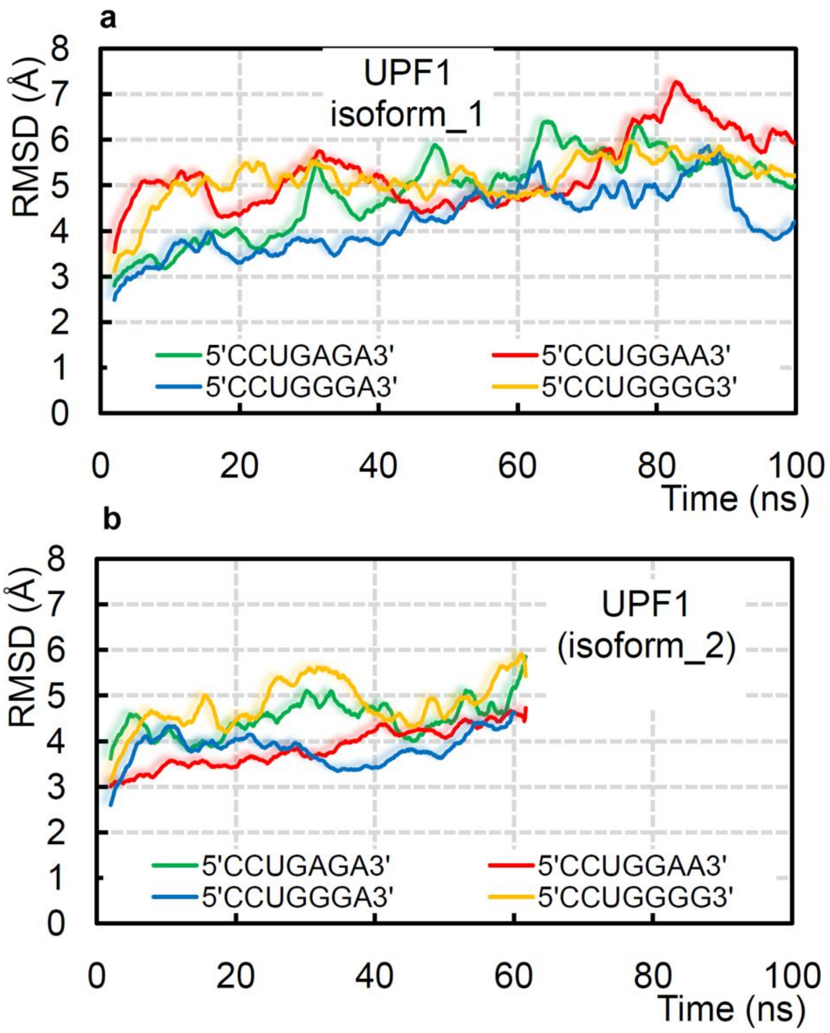

Figure S3. Root-mean-square deviation (RMSD) of atomic positions (excluding hydrogen atoms) for the UPF1 protein in presence of GC-rich mRNA motifs. **(a)** UPF1 isoforom_1 in the presence of different GC-rich mRNA motifs. **b)** UPF1 isoforom_2 in the presence of different GC-rich mRNA motifs. For UPF1 isoform_1, the MD simulation was performed for 100 ns and for UPF1 isoform_2 the MD simulation was performed for ~60 ns. During this time frame it was observed that the UPF1 protein system has reached the equilibrium state in both UPF1 isoforms.

Movies attached as a separate file:

Video S1. The ‘closed conformation’ between 1B-RecA2 domains acquired by the UPF1 (isoform_1) with poly(U) mRNA motif during the 100 ns molecular dynamics. The poly(U) mRNA forming a 4-stack pattern with UPF1 (isoform_1) is highlighted. Color Scheme: mRNA is shown in red color, domain 1B in orange, and RecA2 domain in blue.

Video S2. The ‘open conformation’ between 1B-RecA2 domains acquired by the UPF1 (isoform_2) with poly(U) mRNA motif during the 100 ns molecular dynamics. Color Scheme: mRNA is shown in red color, domain 1B in orange, and RecA2 domain in blue.

Video S3. Mutated $UPF1_{P533T}$ (isoform_1) system lacks the stacking pattern in poly(U) mRNA motif, resulting to an ‘open conformation’ between 1B and RecA2 domains during the 100 ns molecular dynamics. Color Scheme: mutated residue $UPF1_{P533T}$ in green, mRNA is shown in red color, domain 1B in orange, and RecA2 domain in blue.
